# Evaluation of FAST COVID-19 SARS-CoV-2 Antigen Rapid Test Kit for Detection of SARS-CoV-2 in Respiratory Samples from Mildly Symptomatic or Asymptomatic Patients

**DOI:** 10.3390/diagnostics12030650

**Published:** 2022-03-07

**Authors:** Immacolata Polvere, Serena Voccola, Silvia D’Andrea, Lucrezia Zerillo, Romualdo Varricchio, Jessica Raffaella Madera, Romania Stilo, Pasquale Vito, Tiziana Zotti

**Affiliations:** 1 Department of Science and Technologies, University of Sannio, 82100 Benevento, Italy; immapolvere88@gmail.com (I.P.); lzerillo@unisannio.it (L.Z.); jrmadera@unisannio.it (J.R.M.); romstilo@unisannio.it (R.S.); 2 Genus Biotech, University of Sannio, 82100 Benevento, Italy; serenavoccola@gmail.com (S.V.); sil-dand@hotmail.it (S.D.); romualdovar@outlook.it (R.V.)

**Keywords:** SARS-CoV-2, COVID-19, antigenic test, Ag-RDT, RT-qPCR, diagnosis, surveillance

## Abstract

Molecular tests are the gold standard to diagnose severe acute respiratory syndrome coronavirus 2 (SARS-CoV-2) infection but are associated with a diagnostic delay, while antigen detection tests can generate results within 20 min even outside a laboratory. In order to evaluate the accuracy and reliability of the FAST COVID-19 SARS-CoV-2 Antigen Rapid Test Kit (Ag-RDT), two respiratory swabs were collected simultaneously from 501 patients, with mild or no coronavirus disease 2019 (COVID-19)-related symptoms, and analyzed with both the Reverse Transcriptase-quantitative Polymerase Chain Reaction (RT-qPCR) and the FAST COVID-19 SARS-CoV-2 Antigen Rapid Test. Results were then compared to determine clinical performance in a screening setting. We measured a precision of 97.41% (95% CI 92.42–99.15%) and a recall of 98.26% (95% CI 93.88–99.25%), with a specificity of 99.22% (95% CI 97.74–99.74%), a negative predictive value of 99.48% (95% CI 97.98–99.87%), and an overall accuracy of 99.00% (95% CI 97.69–99.68%). Concordance was described by a Kappa coefficient of 0.971 (95% CI 0.947–0.996). Considering short lead times, low cost, and opportunities for decentralized testing, the Ag-RDT test can enhance the efforts to control SARS-CoV-2 spread in several settings.

## 1. Introduction

Since the first reported cases in December 2019, the COVID-19 pandemic has notably challenged public health worldwide and forced governments to impose restrictive measures to contain virus spread and contagion [1,2,3]. The timely detection of positive cases, their isolation and contact tracing are crucial steps in controlling the pandemic, but, on the other hand, the laboratory testing capacity is a limiting factor for the screening of large groups of the population, mainly asymptomatic and repeatedly over time [4]. To date, the World Health Organization (WHO) recommends probe-based Reverse Transcriptase-quantitative polymerase chain reaction (RT-qPCR) testing on RNA extracted from the upper respiratory tract specimens (mainly nasopharyngeal or oropharyngeal swabs) as the gold standard method for the diagnosis of the Severe Acute Respiratory Syndrome Coronavirus 2 (SARS-CoV-2) infection [5,6]. Moreover, the Centers for Disease Control and Prevention (CDC) indicate that rapid point of care serial screening can identify asymptomatic cases and help interrupt the spread of SARS-CoV-2, especially when the transmission risk within a community is notably high [7]. Despite its effectiveness and high sensitivity, RT-qPCR analysis appears poorly sustainable in screening or surveillance settings, as such a testing procedure is time-consuming, requires expensive reagents, qualified personnel, and adequate facilities [4]. Therefore, there is a critical demand for alternative approaches such as antigen-detection rapid diagnostic tests (Ag-RDTs), which can detect the presence of the SARS-CoV-2 virus itself in respiratory samples within 20 min even outside a laboratory [8]. Plenty of tests have recently been developed and many of them are now commercially available [9]. However, claims of the antigen tests’ accuracies by their manufacturer usually do not hold true in real world settings, that is among mildly symptomatic or asymptomatic individuals, although many findings suggest that pre-symptomatic or asymptomatic transmission accounts for a high proportion of the spread of COVID-19 [4,10,11,12]. The aim of the study was to evaluate the clinical performance of the FAST COVID-19 SARS-CoV-2 Antigen Rapid Test (Ag-RDT), an immunocapture-based device intended to detect SARS-CoV-2 Nucleocapsid Proteins from nasal specimens, and to measure the agreement with the RT-qPCR results by a comparative analysis of respiratory swabs from 501 patients with mild or no COVID-19-related symptoms.

## 2. Materials and Methods

### 2.1. Population Study and Informed Consent

The study was carried out between October 2020 and January 2021 at the private diagnostic laboratory Centro Delta (Apollosa, Benevento, Italy) on a cohort of persons who spontaneously attend the Centro Delta to undergo a test for the diagnosis of COVID-19. People went to the diagnostic laboratory for different reasons: either because of the appearance of symptoms, or due to previous contact with patients who had tested positive, or for simple routine screening. Patients were informed about the ongoing study and, after signing informed consent, were asked to answer a COVID-19 symptom-based screening questionnaire and undergo a second swab for comparative purposes.

### 2.2. Sample Collection

Trained personnel collected two swabs from each patient. One single cotton swab was used for sampling from the back of the throat and subsequently from the deep nasopharynx (oropharyngeal and nasopharyngeal swab) and analyzed by RT-qPCR. Soon after, a second swab was used to collect a nasal specimen for the Ag-RDT. The two swabs were individually put in sterile tubes, labeled and stored at 4 °C until analysis. RT-qPCR and Ag-RDT testing analysis were performed within 2 h from collection with the appropriate safety precautions. Sample collection, demographic data collection, antigenic testing and molecular analysis were carried out by different operators.

### 2.3. Study Design

**Figure 1:**
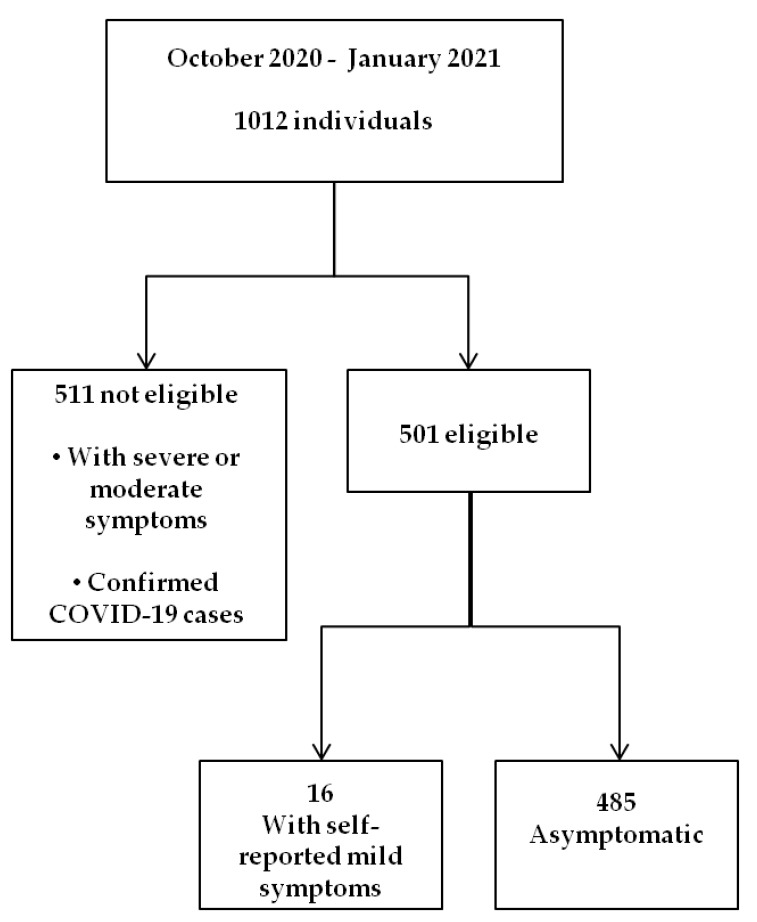
Flow chart summarizing the study design. Over a period of four months, 1012 individuals were tested for SARS-CoV-2 infection. Eligible patients for the comparative study were 501 individuals declaring mild or no COVID-19 related symptoms and not previously diagnosed as COVID-19-cases.

The study was designed according to the Declaration of Helsinki and approved by the Institutional Review Board of Consorzio Sannio Tech (n. 01/2020). Inclusion criteria: patients at any age with no or mild symptoms related to COVID-19. For minors, parental consent was required. Exclusion criteria: Patients with severe or moderate symptoms or patients already diagnosed as COVID-19 positive but not yet recovered. Over the study period, more than one thousand people asked for a COVID-19 diagnostic test, but only 501 were eligible for the comparative study (Figure 1). The exclusion of confirmed COVID-19 cases is based on the requirement to evaluate the performance of the F-Ag-RD in a screening/surveillance setting. Illness severity was determined according to the NIH COVID-19 Treatment Guidelines [13]. On the basis of self-reported symptoms, participants were classified as “asymptomatic” when it was declared that they had no symptoms that were consistent with COVID-19 or “mildly symptomatic” when declared with at least one among the following COVID-19 infection-related signs: fever, cough, sore throat, malaise, headache, muscle pain, nausea, vomiting, diarrhea, loss of taste and smell. Shortness of breath, dyspnea, any evidence of a lower respiratory disease or oxygen saturation (SpO2) lower than 94% in room air, were considered to be signs of moderate or severe illness. Therefore, patients with such manifestations have been excluded from the study.

### 2.4. Sample Preparation and RT-qPCR

Molecular detection of SARS-CoV-2 was performed in the CFX96 Touch Real Time PCR Detection System (Bio-Rad Laboratories) with the dual probe-based IVD-validated SARS-CoV-2 Real Time kit (Nuclear Laser Medicine s.r.l) according to the manufacturer’s instructions, as described elsewhere [14]. Under a laminar flow-cabinet, respiratory specimens were treated with 350 μL of a provided Extraction Buffer by vigorously vortexing for 1 min, followed by a heat inactivation step at 95 °C for 10 min. Then, samples were briefly spun down and 13 μL of extract was used for multiplex RT-qPCR. An internal control was used as amplification control and the target gene E was used as a common feature of beta-coronavirus. According to the manufacturer’s instructions, a sample was positive for SARS-CoV-2 infection when at least one between N and RdRP/Hel was specifically amplified at or before the Cycle Threshold (Ct) of 40. In particular, valid samples have been diagnosed according to Table 1.

### 2.5. Ag-RDT

The FAST COVID-19 SARS-CoV-2 Antigen Rapid Test kit is a colloidal gold—immunocapture-based device manufactured by JOYSBIO Biotechnology Co. LTD (Tianjin, China), lot number 2020090804 (exp. date 9 June 2022) and branded by Tecno Bios srl (Apollosa, Italy), intended to detect the presence of SARS-CoV-2 Nucleocapsid Proteins in nasal specimens collected from patients. The Ag-RDT was carried out according to the manufacturer’s instructions. Reading of the cassette was performed within 20 min after sample loading.

### 2.6. Recombinant Protein Production and Purification

Recombinant proteins were produced in bacteria as previously described [15]. A cDNA encoding SARS-CoV-2 Nucleocapsid protein gene (Wuhan, Accession: QHD43423.2) was cloned into the pET28a vector. The His-tagged recombinant SARS-CoV-2 N protein was obtained by inducing expression in competent *E. coli* BL21 cells with 1 mM Isopropil-β-D-1-tiogalattopiranoside (IPTG), extracted by alternating sonication with freeze/thaw cycles and then purified with Nickel-beads. The purified N concentration was measured with a NanoDrop Microvolume Spectrophotometer (Thermo Fisher Scientific, Carlsbad, CA, USA) and, in order to determine the limit of detection (LOD) of the Ag-RDT, different amounts of the recombinant N protein dissolved in 100 μL of the provided extraction buffer by serial dilutions were added to the sample pad of the testing cassette.

### 2.7. Statistical Analysis

The performance of the Ag-RDT was evaluated using JMP Trial 15. Specificity and sensitivity were calculated considering the RT-qPCR as the reference method. The overall percentage of the agreement and the Cohen coefficient (k) were used to determine the accuracy of the test. Chi-square/Fisher’s exact test were performed to assess statistical association of SARS-CoV-2 diagnosis with demographic data, symptoms and the Ag-RDT results. A *p*-value lower than 0.05 was considered statistically significant.

## 3. Results

A total of 501 individuals, 204 (40.7%) females and 297 (59.3%) males, with mild or no COVID-19-related symptoms were included in this comparative study. The age of participants ranged from 6 to 90 years old, with a median age of 43.9. Population features are represented in Table 2.

Double swabs (one from the oropharynx and nasopharynx and one only nasal) were collected at the same time from eligible patients over a period of four months. Different collection sites were used because, as specifically indicated by the manufacturer’s instructions, the JOYSBIO Ag-RDT is intended to detect SARS-CoV-2 in nasal specimens.

485 (96.8%) participants declared no symptoms, whereas 16 (3.2%) declared at least one among the following symptoms: fever > 37.5 °C; cough; sore throat; malaise; headache; muscle pain; nausea; vomiting; diarrhea; loss of taste and smell.

**Figure 2:**
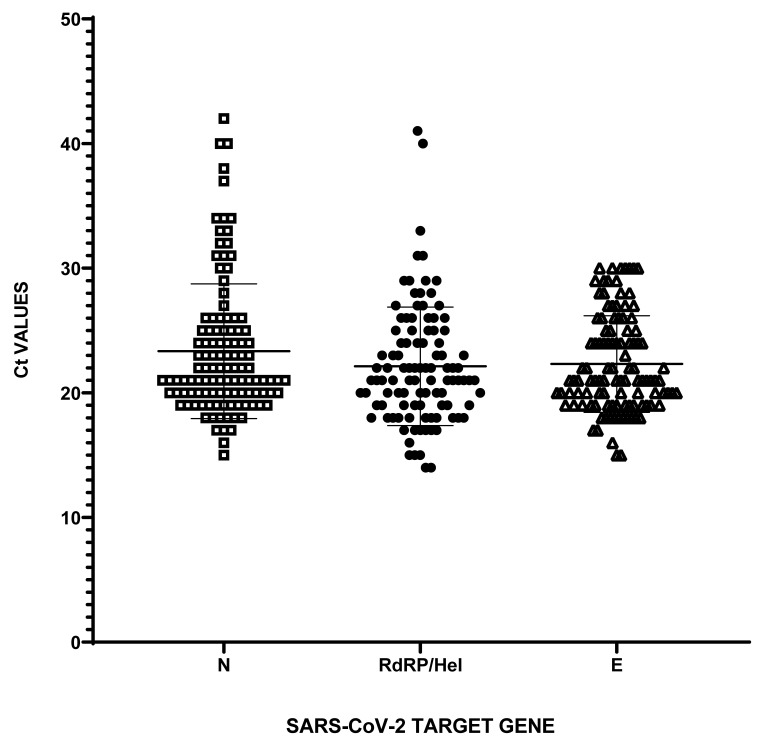
RT-qPCR amplification results of positive samples. Ct values for probe-based RT-qPCR amplification of N, RdRP/Hel and E genes in 115 SARS-CoV-2-positive samples.

According to the RT-qPCR, 386 (77.1%) samples were a negative result and 115 (22.9%) were a positive result. The average cycle threshold (Ct) values for positive cases were 22.31 ± 3.87 (min Ct 15; max Ct 30) for the E gene; 22.12 ± 4.74 (min Ct 14; max Ct 41) for the RdRp/Hel gene; and 23.33 ± 5.37 (min Ct 15, max Ct 42) for the N gene (Figure 2).

**Figure 3:**
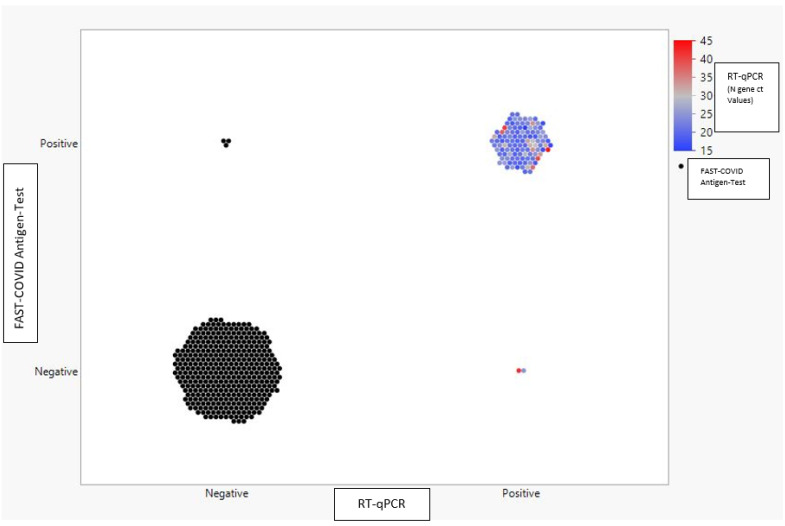
Comparison of RT-qPCR and antigenic test results. Graphical distribution of 501 SARS-CoV-2-positive and -negative samples detected through FAST COVID Ag-RDT respect to Ct values for the N gene resulting from RT-qPCR analysis. All colored dots are representative of SARS-CoV-2-positive samples diagnosed as described in the Materials and Methods section. Red dots represent samples with very late or no amplification of N (Ct > 40) but positive for RdRP/Hel gene amplification.

Out of the 115 positive samples diagnosed through RT-qPCR, the Ag-RDT detected 113 positive samples and 2 negatives (considered false negatives), whereas out of the 386 negative samples, the Ag-RDT detected 383 consistent samples and 3 false positives (Figure 3).

Among positive samples to RT-qPCR test, 36 (31.30%) showed mean Ct values for the analyzed viral genes ≤ 20, 66 (57.39%) showed a mean Ct > 20 and ≤ 30, and 13 (11.30%) had a mean Ct value ≥ 30 and <40, indicating that, using Ct values as a proxy of viral load, more than half of the positives identified have a high (mean Ct ≤ 20) or medium-high (20 > Ct ≥ 30) viral load. Interestingly, one false negative sample showed medium Ct values (N: 25; E: 24; RdRp/Hel: 25; mean CT values: 24.67) and the other had high Ct values (N: 42; E: 30; RdRp/Hel: 29; mean CT values: 33.67), confirming that the Ag-RDT is able to detect SARS-CoV-2 antigens from specimens with a low copy number of viral genomes but has a lower sensitivity compared to RT-qPCR.

Overall, the studied population showed a positivity rate of 22.95% (95% CI 19.34–26.89%). Using the RT-qPCR as a reference method, the Ag-RDT detected 113 SARS-CoV-2 actively infected patients out of 115 confirmed positives with a recall/sensitivity of 99.22% (95% CI 97.74–99.74%) and a precision/positive predictive value (PPV) of 97.41% (95% CI 92.42–99.15%). Specificity was 98.26% (95% CI 93.88–99.25%) and negative predictive value (NPV) was 99.48% (95% CI 97.98–99.87%). Overall, we observed a diagnostic accuracy of 99.00% (95% CI 97.69–99.68%) and an F1-score (evaluated as $2\;\cdot \;\frac{\left(\mathrm{precision}\;\cdot \;\mathrm{recall}\right)}{\left(\mathrm{precision}+\;\mathrm{recall}\right)}\;)\;\mathrm{of}\;97.84\%$. The general agreement between the RT-qPCR and the Ag-RDT was 97.18% (k = 0.9718; 95% CI: 0.94–0.99).

Next, we evaluated the association of SARS-CoV-2 diagnosis through the RT-qPCR with demographic features, with symptoms and with the Ag-RDT results (Table 3). As assessed by Chi-square/Fisher’s Exact test, positive diagnosis of SARS-CoV-2 infection with either the RT-qPCR or the Ag-RDT is not significatively associated with sex and age. Also, positive or negative diagnosis was not statistically associated with manifestation of symptoms, suggesting that both methods similarly detect infected individuals even without or before the onset of symptoms. Conversely, association between the RT-qPCR and Ag-RDT results was statistically verified.

**Figure 4:**
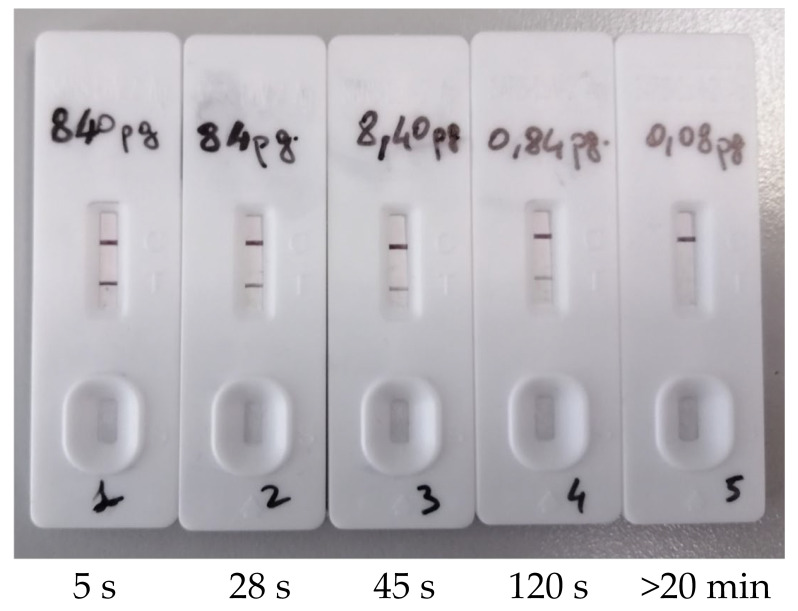
Assessment of limit of detection for the Ag-RDT. Serial dilutions of recombinant SARS-CoV-2 Nucleocapsid (N) protein have been used to assess the lowest concentration detectable in the antigenic test. The time of appearance of the colored band in the window of the test line is also reported. Results on the cassettes have been read within 20 min.

In addition, serial dilutions of the purified recombinant SARS-CoV-2 N protein were used to determine the lowest concentration that yielded positive results in the Ag-RDT. As shown, 0.84 pg dissolved in 100 μL of the kit extraction buffer is the minimum antigen amount detectable for the FAST COVID-19 Antigenic test device (Figure 4). Since the molecular weight of His-tagged recombinant N is 46466.50 Dalton, we have estimated that the number of recombinant N molecules present in the Ag-RDT LOD of 0.84 pg is about 1.09 × 10^7^. Assuming that each SARS-CoV-2 virion contains ~10^3^ molecules of N [16], the minimum number of virions (and, by consequence, of genome copies) recognizable by the Ag-RDT is approximatively 1.09 × 10^5^ per mL.

## 4. Discussion

In the present report, the Ag-RDT showed high recall/sensitivity (98.26%), high specificity (99.22%), high positive predictive value (PPV)/precision (97.41%), and an overall accuracy of 99.00%. These results are far above both the minimum and desirable requirements of the WHO, that are, respectively, ≥80% and ≥90% for sensitivity and ≥97% and >99% for specificity [17]. We also could measure a high degree of concordance respect to RT-qPCR, used as the reference method, with a Kappa coefficient of 0.971. Such evidences suggest that this kit is able to accurately identify the true positives for medium-low prevalence disease and could be used to verify RT-qPCR results in screening settings, even when illness is mild or not yet overt [18].

One main limitation of our study is that, although the comparative study was performed on gold standard specimens for each type of the tests [19], we used two different sampling sites (oropharyngeal and nasopharyngeal cavities for molecular analysis and nasal walls for antigen analysis), which might exhibit different viral loads. Secondly, illness severity has been only assessed at the time of sampling, based on what the patient declared, that is, we have no information about the course of the disease after the collection.

Since the beginning of virus spread, there are controversial views about the infectiousness of patients showing no or mild COVID-19 related symptoms. Recently, many reports have demonstrated that asymptomatic patients could be carriers of high viral loads, as assessed by low Ct values for viral gene amplification through RT-qPCR, representing thereby the hidden part of the iceberg in terms of contagion propagation [12,20,21]. On the other hand, “naїve” Ct values can be affected by several variables (e.g., sampling, RNA integrity and purity after extraction, batch effect) and, without comparing them with a standard curve of reference materials, cannot be considered an absolute correlate of viral load [22]. As others demonstrated that illness severity is not strictly related to viral load and, by consequence, to contagiousness, we excluded patients with severe or moderate symptoms or COVID-19-confirmed cases from our study, basing this choice on the will to evaluate the performance of the Ag-RDT on “first entries” in a screening/surveillance setting. Interestingly, plenty of studies have reported the evaluation of several Ag-RDTs among both symptomatic and asymptomatic individuals [9], with performance parameters that, for most Ag-RDTs, appear notably better when analysis is carried out in the early phase of disease or on samples either with lower RT-qPCR Ct values or from patients with moderate or severe illness [23,24,25].

The FAST COVID-19 SARS-CoV-2 Antigen Rapid Test manufactured by JOYSBIO has been evaluated in three distinct reports [26,27,28].

Cubas-Atienzar et al. have determined the limit of detection of the Ag-RDT in different matrices and they found that it ranges between 2.2 × 10^5^ genome copy number/mL evaluated through direct viral culture and 2.7 × 10^8^ genome copy number/mL evaluated through the use of dry swabs [26]. In our evaluation study, we observed a LOD of 8.4 pg/mL assessed by using recombinant His-tagged N protein, that we have inferred would correspond approximately to 1.09 × 10^5^ virions/mL. Assuming that the ratio of virions to genome copies is virtually ~1, our LOD estimation is slightly lower than the LOD measured by Cubas-Atienzar et al., through direct viral culture [26]. However, we cannot exclude that, in the determination of the LOD, the use of the purified recombinant antigen instead of the infectious viral particles has led to either an underestimation or an overestimation of the minimum value detectable by the antigen test.

The no-profit foundation FIND evaluated the Ag-RDT on 265 patients enrolled either as suspected COVID-19 cases, with or without symptoms, or as part of routine medical care [27]. The FIND report shows a clinical specificity of 99.10% and a clinical sensitivity of 70.50%, which, when calculated with respect to positive samples with Ct ≤ 25 for N, reach 91.30% [27]. In our data, we were unable to identify a causal relationship between the Ct values observed in the positive samples and the antigen test result, for two main reasons: (1) we used two different sampling sites which might present different viral loads; (2) the only two positive samples not detected by the Ag-RDT had two diverse mean Ct values for the viral genes (33.67 considered weakly positive and 24.67 considered positive). However, another explanation of the observed discrepancies could be due to the fact that antigenic diagnostics for SARS-CoV-2 continually advances, with new pairs of detecting/capturing antibodies continually being tested. Since manufacturers usually do not report the clones of antibodies used in their Ag-RDTs, it may well be that their performances improve thanks to the use of increasingly efficient antibodies. This implies that in the analysis of the performances of Ag-RDTs, it should be mandatory to indicate the lot number of the tested devices.

In the paper by Homza et al., the Ag-RDT by JOYSBIO was evaluated on 225 patients in contrast to the RT-qPCR, with acceptable performance parameters. Nevertheless, when the authors corrected such parameters on the virus viability evaluated on discordant samples, they were remarkably improved (specificity resulted 98.80%, sensitivity/recall 92.90%, NPV 97.70%, 97.30% accuracy, whereas PPV was 96.30% and did not change), suggesting that evaluation of the reliability of SARS-CoV-2 diagnostic tests should take virus viability into consideration [28]. Indeed, RT-qPCR analysis and other NAATs are able to detect amount of viral RNA that cannot be cultured, indicating that the presence of viral nucleic acid does not always indicate contagiousness [4,29]. The higher sensitivity of antigen tests recorded when viral culture is used as a reference instead of RT-qPCR, points out to the fact that the detection capability is strictly related to viral load, as discussed in other reports [29,30].

Additionally, as reviewed by Cevik et al. [31], the SARS-CoV-2 viral load appears to peak in the first week of illness in the upper respiratory tract, when symptoms are still absent, or mild. In our hands, the F1 score of the FAST COVID-19 SARS-CoV-2 Antigen Rapid Test is 97.84% indicating that, although we cannot say for sure, the group of participants in our study mostly included recently infected patients who were in a pre-acute or acute phase of the disease, that is when the viral load grows exponentially but the symptoms are not yet evident. Therefore, our study is consistent with the idea supported by previous papers that the viral load is clearly the most important factor that influences sensitivity for SARS-CoV-2 antigen testing [31]. The practice of testing previously confirmed COVID-19 patients for the evaluation of rapid test performances can in fact leads to an overestimation of false negatives, not so much because the test is poorly sensitive, but because, probably after the acute phase of the first seven to eight days, the virus moves to the lower respiratory tract, while in the upper tract only non-infectious viral residues remain [31].

Ultimately, although less sensitive than molecular approaches, Ag-RDTs have helped rapid diagnosis of asymptomatic or pauci-symptomatic positive individuals in settings with high risk of transmission, or where repeated testing is required. Also, they find wide and successful applications in the screening of large “circulating” communities such as school or university campuses [32], emergency rooms [25], and, more generally, in settings of low resources or low testing capacity [24]. However, one should keep in mind that since the FAST COVID-19 SARS-CoV-2 Ag-RDT had an approximate LOD of 10^5^ viral particle/mL to score a positive sample and the readout generally is not quantitative, this rapid test cannot give exact information about how much time has elapsed between infection of an asymptomatic patient and a positive result. It is also important to underline that the performance parameters of antigen tests, such as LOD, precision/recall scores, and the “timeliness” of detection of infective individuals, should be ex novo evaluated whenever a novel variant of the virus is prevalent in the population.

On this basis, proper evaluation, application and implementation of different tests, and diagnostic strategies, such as sample pooling, molecular, antigenic, salivary and serological tests, as well as the careful interpretation of results, represent the most effective way to optimize public health control measures and contain both actual and future pandemics [4,14,33,34,35,36,37,38].

## Figures and Tables

**Table 1:** Scheme for the diagnosis of SARS-CoV-2 positivitiy by RT-qPCR.

N	RdRP/Hel	SARS-CoV-2 Infection
Ct > 40 or absent	Ct > 40 or absent	Not detected
Ct ≤ 40	Ct > 40 or absent	Detected
Ct > 40 or absent	Ct ≤ 40	Detected

**Table 2:** Demographic data and symptoms’ manifestations of volunteers participating to study.

			Gender						
Age	No.	%	Female	%	Male	%	Symptoms	No.	%
0–20	34	6.79	12	2.40	22	4.39	Mild symptoms	16	3.19
21–40	194	38.72	71	14.17	124	24.75	Asymptomatic	485	96.81
41–60	174	34.73	81	16.17	92	18.36	TOTAL	501	100.00
61–80	80	15.97	33	6.59	47	9.38			
81–100	12	2.40	5	1.00	7	1.40			
N/A	7	1.40	2	0.40	5	1.00			
TOTAL	501	100.00	204	40.72	297	59.28			

**Table 3:** Contingency table showing the association of the RT-qPCR results with age, gender, symptoms and the results of the rapid test (two last lines) evaluated by Chi-square/Fisher’s exact test. The *p*-value from a Fisher’s test is exactly determined and quantifies the significance of the deviation from the hypothesis of non-association (null hypothesis). Statistically significant *p* < 0.05.

Features		Overall (*n* = 501)		RT-qPCR Positive (*n* = 115)			RT-qPCR Negative (*n* = 386)			*p*-Value *	Ag-RDT Positive *(n* = 116)			Ag-RDT Negative (*n* = 385)			*p*-Value *
		No.	%	No.	%	% within the Same Group	No.	%	% within the Same Group		No.	%	% within the Same Group	No.	%	% within the Same Group	
**Gender**	Male	297	59.28	65	12.97	21.89	232	59.28	78.11	0.5174	64	12.77	21.55	233	46.51	78.45	0.3324
	Female	204	40.72	50	9.98	24.51	154	30.74	75.49		52	10.38	25.49	152	30.34	74.51	
**Age Group**	0–20	34	6.79	8	1.60	23.53	26	5.19	76.47	0.8874	8	1.60	23.53	26	5.19	76.47	0.9588
	21–40	194	38.72	41	8.18	21.13	153	30.54	78.87		42	8.38	21.65	152	30.34	78.35	
	41–60	174	34.73	43	8.78	24.71	131	26.15	75.29		44	8.78	25.29	130	25.95	74.71	
	61–80	80	15.97	18	3.59	22.50	62	12.38	77.50		18	3.59	22.50	62	12.38	77.50	
	81–100	12	2.40	4	0.60	33.33	8	1.60	66.67		3	0.60	25.00	9	1.80	75.00	
	N/A	7	1.40	1	0.20	14.29	6	1.20	85.71		1	0.20	14.29	6	1.20	85.71	
**Symptoms**	Mild symptoms	16	3.19	4	0.80	25.00	12	2.39	75.00	0.7691	4	0.80	25.00	12	2.40	75.00	0.7709
	Asymptomatic	485	96.81	111	22.16	22.89	374	74.65	77.11		112	22.36	23.09	373	74.45	76.91	
**Ag-RDT results**	Positive	116	23.15	113	22.55	97.41	3	0.60	2.59	<0.001 *							
	Negative	385	76.85	2	0.40	0.52	383	76.85	99.48								

* Evaluated by Fisher’s Exact test.

## Data Availability

Data are available upon request from the corresponding author.

## Abbreviations

RT-qPCR: Reverse Transcriptase-quantitative Polymerase Chain Reaction; COVID-19: Coronavirus Disease 2019; SARS-CoV-2: Severe Acute Respiratory Syndrome Coronavirus 2; Ag-RDT: Antigen-detection Rapid Diagnostic Test; NAATs: Nucleic Acid Amplification Tests; Ct: Cycle Threshold.
